# Editorial: Neuronal ensembles and memory engrams: Cellular and molecular mechanisms

**DOI:** 10.3389/fnbeh.2023.1157414

**Published:** 2023-02-28

**Authors:** Leslie A. Ramsey, Eisuke Koya, Michel C. van den Oever

**Affiliations:** ^1^Behavioral Neuroscience Research Branch Intramural Research Program, National Institute on Drug Abuse (NIDA), National Institutes of Health (NIH), Baltimore, MD, United States; ^2^School of Psychology, Sussex Neuroscience, University of Sussex, Falmer, United Kingdom; ^3^Department of Molecular and Cellular Neurobiology, Center for Neurogenomics and Cognitive Research, Amsterdam Neuroscience, Vrije Universiteit Amsterdam, Amsterdam, Netherlands

**Keywords:** learning and memory, synaptic engram, immediate early gene (IEG), Fos, Arc, mGRASP

One of the fundamental questions of neuroscience is how the brain can create, store, and retrieve memories. In the early part of the twentieth century, Karl Lashley attempted to shed light on this question using cortical lesion studies. He concluded that complex behavior relies on both local and distributed storage and retrieval mechanisms in the brain. Lashley (1931) acknowledged that multiple, dispersed brain regions were necessary to enable complex behavior, a principle he referred to as mass action of cerebral function. The search for the mechanistic substrates of memory, what Richard Semon called the “engram” has continued into the present day (Semon, 1921). Later, Hebb (1949). pioneered the idea of neuronal ensembles, which he referred to as “cell assemblies,” or small populations of sparsely distributed neurons active in response to a specific salient stimulus. Another theory put forth by Hebb was the idea that learning occurs *via* strengthening of synaptic connections between neurons, or synaptic (Hebbian) plasticity. Hebb, Lashley, and their contemporaries had access to a limited toolkit and relied mostly on lesion studies and clinical case studies to test their theories. Fortunately, in the past two decades there has been a renaissance in tools and technology available to identify, characterize, and manipulate neuronal ensembles and engrams (Koya et al., 2009; Kim et al., 2011; Choi et al., 2018; DeNardo et al., 2019; Matos et al., 2019). The advent of these tools has led to an explosion of research that is beginning to uncover the cellular and molecular mechanisms by which memories are encoded and retrieved. This Research Topic contains five papers that further our understanding, both empirically and theoretically, of the cellular and molecular mechanisms within neuronal ensembles that support the engram, using both established methods and cutting-edge technology, as well as incorporating new statistical approaches.

Josselyn et al. previously demonstrated that neurons made more excitable prior to fear conditioning are more likely to be allocated into a fear memory ensemble and are preferentially reactivated during memory retrieval (Han et al., 2007; Yiu et al., 2014). In this topic, Cho et al. demonstrate that neurons in the lateral amygdala that are active during the formation of a fear memory ensemble are less likely to be reactivated during memory recall and required for memory expression when retrieval is preceded by retraining mice 24 h after initial fear conditioning. Interestingly, when retraining occurred only 6 h after fear conditioning, the initial fear memory ensemble remained essential for subsequent memory recall, suggesting a form of “co-allocation.” The effect was specific to retraining; recall alone did not have the same effect. Their findings imply that there may be separate allocation mechanisms that drive initial learning and relearning. Sortman et al. use the Daun02 inactivation method in Fos-LacZ transgenic rats to demonstrate that Fos-expressing neuronal ensembles in the prelimbic cortex mediate both recently acquired and well-trained cocaine self-administration. These findings build on their previous work showing a critical role for the self-administration ensemble in cocaine seeking (Kane et al., 2021).

Koutlas et al. used a method known as targeted recombination in activated populations (TRAP) by means of the TRAP2 transgenic mouse line (DeNardo et al., 2019) to identify a neuronal ensemble within the VTA that is activated by social stress. The social stress ensemble was a relatively small population (~11%) of cells, and heterogeneous in terms of cell types. Furthermore, the authors discovered that the social stress-activated neurons are more excitable than surrounding non-ensemble neurons, even when recorded a month after the initial exposure to stress. Such persistent changes in excitability are remarkable given that stress exposure was acute. Just one excellent example of what we can begin to uncover with the availability of tools such as the TRAP2 system. An interesting question for future research is to identify the molecular mechanism by which excitability is heightened in the social stress ensemble neurons. Murthy et al. employed the mammalian GFP reconstitution across synaptic partners (mGRASP) system (Kim et al., 2011) to identify synaptic connections between activated CA1 and CA3 ensemble neurons. They find that the Arc-TRAP system allows efficient synaptic labeling compared to the Fos-tTA system, most likely driven by the difference in permanent vs. transient labeling, respectively. The authors suggest that the possibility to combine mGRASP technology with two-photon microscopy will enable longitudinal monitoring of synapses between ensemble cells, bringing us closer to identifying the “synaptic engram.” This advance allows ensemble and engram researchers to empirically test theories about Hebbian synaptic plasticity and associative learning that have been part of theoretical dogma for decades.

Finally, Körber and Sommer review studies on neuronal ensembles active in pursuit of drug and non-drug rewards, identified using the immediate early gene *c-fos*. A particular emphasis is placed on studies of alcohol-related seeking ensembles. The authors discuss the use of graph theory-based network science to identify ensembles and to infer which brain regions form higher order neuronal ensembles, or meta-ensembles. This approach represents a major advance in our overall understanding of brain-wide ensemble dynamics because it helps us to overcome the limitation of studying ensembles by characterizing a single brain region at a time.

The broad range of brain regions, sensory modalities, types of learning and various engram labeling methodologies displayed in this topic show that there are many approaches to answering the essential question of how the brain can learn, store, and retrieve information. An important topic for future research is to investigate the identity and stability of the neurons that harbor engrams. For instance, are memories allocated to dedicated neurons that are meant to act as information storage units from birth onwards? If true, it will be relevant to determine which proportion of neurons in the brain is dedicated for this purpose and whether these cells can be distinguished based on their genetic, physiological, and morphological properties. Recent studies demonstrate that after memory allocation, memory-supporting neuronal ensembles can be stable over time. In particular, cortical ensembles remain essential for memory expression several weeks after memory acquisition (Kitamura et al., 2017; Matos et al., 2019; Visser et al., 2020). Insight into the molecular and cellular processes that occur within these cells to consolidate and retrieve the memory is crucial for understanding mechanisms that contribute to memory persistence. Vice versa, it is of relevance to investigate which processes within neuronal ensembles mediate fading, or forgetting, of memories. Enhanced synaptic strength has recently been described between cortical ensemble neurons and disruption of this connectivity impaires remote memory expression (Lee et al., 2023). This indicates that a lasting synaptic engram supports memory retention, however, the molecular pathways supporting a synaptic engram remain to be elucidated.

Furthermore, longitundinal imaging techniques, such as two-photon microscopy and calcium imaging in freely moving animals, provide a plethora of possibilities to increase our understanding of the spatiotemporal dynamics of neuronal ensembles and the relationship between learning-induced neuronal activity and immediate early gene-based labeling methods. For technical reasons, most of these studies have thus far been performed in superficial brain structures, leaving important questions about deep brain structures involved in information storage largely unaddressed. For instance, it is poorly understood how reward-associated information is real-time processed by neuronal ensembles in the striatum. Finally, although opto- and chemogenetic manipulation techniques have provided invaluable evidence for the causal involvement of neuronal ensembles in memory processing using animal models, it remains a challenge to translate ensemble interventions to the clinic. With this objective in mind, it is also of utmost importance to acquire detailed insight into the learning-induced physical adaptations (i.e., the engram) that develop in memory-encoding neuronal ensembles. Specifically, identification of neurobiological substrates that are unique to a specific engram, e.g., drug reward or traumatic memory, holds promise for development of therapeutic intervention options.

## Author contributions

LR, EK, and MO contributed to the writing of this editorial. All authors contributed to the article and approved the submitted version.
